# A case report: Infection-related glomerulonephritis and mantle cell lymphoma due to mycobacterium avium complex infection

**DOI:** 10.1097/MD.0000000000035620

**Published:** 2023-12-29

**Authors:** Yiqi Huang, Li Xia, Weigang Shen, Tianxiao Fu

**Affiliations:** a Department of Nephrology, Shaoxing Second Hospital, Shaoxing, Zhejiang, China; b Department of Traditional Chinese Medicine, The First Affiliated Hospital, College of Medicine, Zhejiang University, Hangzhou, Zhejiang, China.

**Keywords:** infection-associated glomerulonephritis, mantle cell lymphoma, mNGS, mycobacterium avium complex, peritonitis

## Abstract

**Rationale:**

Mycobacterium avium complex (MAC) infection is common in lung, liver and skin. However, MAC presenting with peritonitis is uncommon and is particularly rare in immunocompetent patients. We report a case of infection-associated glomerulonephritis and mantle cell lymphoma caused by peritonitis due to MAC.

**Patient concerns:**

We report a case of a 73-year-old elderly man with fever and abdominal pain for 2 days and gradually developed anuria, ascites, and abdominal lymphadenopathy.

**Diagnoses:**

The initial diagnosis was peritonitis and acute renal failure. There was no significant relief of symptoms after empirical anti-infective therapy and hemodialysis. infection-associated glomerulonephritis, mantle cell lymphoma, and peritonitis due to MAC were diagnosed by renal biopsy, abdominal lymph node biopsy, and metagenomics next-generation sequencing.

**Interventions:**

The patient received empirical antibiotic therapy, hemodialysis, and anti-MAC therapy.

**Outcomes:**

Unfortunately, the patient eventually died of septic shock after the 21st day of admissiom.

**Lessons:**

Early diagnosis of MAC infection is essential. When the cause of fever is unknown, metagenomics next-generation sequencing can be considered.

## 1. Introduction

Mycobacterium avium complex (MAC) is classified a common and slow-growing nontuberculous mycobacteria (NTM), which is widespread in the natural environment.^[1–3]^ Recently, the incidence of MAC is increasing annually, and the mortality rate associated with MAC infections is increasing year by year.^[4,5]^ As an opportunistic pathogen of humans, MAC usually occurs in immunocompromised patients, especially with HIV, cirrhosis, continuous ambulatory peritoneal dialysis, and solid organ transplantation.^[4]^ Disseminated MAC infection can involve multiple organs other than lungs, including liver, intestine, and bone marrow.^[6,7]^ However, peritonitis due to MAC (PMAC) is a rare but serious and life-threatening condition. To date, 51 cases of PMAC have been reported,^[8]^ including 21 of those case died and only 1 case developed infection-associated glomerulonephritis (IRGN),^[9]^ but mantle cell lymphoma (MCL) caused by PMAC has not been described before. In this report, we first reported a rare case of IRGN and MCL induced by PMAC in an immunocompetent patient, which will help hematologists and nephrology physicians to further understand PMAC and provide valuable clues for early clinical diagnosis and treatment.

## 2. Case presentation

A 73-year-old male patient was admitted to our hospital with a history of fever and abdominal pain for 2 days. There was no complaint of cough, diarrhea and bloody stools. He had been diagnosed to have COVID-19 with asymptomatic approximately 2 months before.

On admission, his vital sign was the following: body temperature of 38.3°C, regular pulse rate is 99/minutes, respiratory rate is 20/minutes, and blood pressure is 139/77 mm Hg. Upon physical examination, he had bilateral coarse breath sounds, diffuse abdominal pain.

Laboratory examination demonstrated the following: white cell count, 15.3 × 109/L (3.5–9.5 × 109/L), hemoglobin 12.5 g/dL (11.5–15 g/dL), albumin 27.4 g/L (40–55 g/L), serum creatinine 189 umol/L (57–111 umol/L), hypersensitive C-reactive protein 89.3 mg/L (0–5 mg/L), erythrocyte sedimentation rate 85 mm/hours (0–15 mg/L), procalcitonin 0.14 ng/mL (0–0.10 ng/mL), lactic dehydrogenase 357 U/L (91–245 U/L). All other clinical examination results were unremarkable.

A computed tomography scan of the thoracoabdominal showed a small pleural and abdominal effusion. Abdominal color ultrasound detected mild splenomegaly and abdominal effusion. Three days after admission, his average 24-hour urine volume was only about 550 mL daily. With the clinical data, he was tentatively diagnosed as having peritonitis and acute kidney injury, and empirical anti-infective treatment (piperacillin-tazobactam, 100 mg/kg/day) for 6 days.

However, the patient’s condition quickly deteriorated with persistent fever (38.0–39.3 °C), anuric state, abdominal pain and distension, which indicated the ineffectiveness of empirical anti-infective therapy. On day 6th of admission, the antibiotic treatments was intensified and changed to meropenem (3 g/day). Over the following days, we once again conducted laboratory test to establish the etiology of the patient’s symptoms and the outcomes has significantly worse (Table 1). The abdominal computed tomography showed the diffuse intestinal canal wall thickening and edema and abdominal effusion as well as lymphadenopathy.

**Table 1:** Clinical and laboratory characteristics from the beginning to the end of treatment.

Measure	Day 0	Day 6	Day 9	Day 10	Day 12	Day 14	Day 17
White cell count (×10^9^/L)	15.3	18.9	-	21.5	-	19.8	-
Hemoglobin (g/dL)	12.5	11.0	-	7.9	-	7.3	-
Albumin (g/L)	27.4	25.7	-	21.6	-	19.7	-
Serum creatinine (umol/L)	189	326	-	314	-	275	-
Hypersensitive C-reactive protein (mg/L)	89.3	158.6	-	192.3	-	246.8	-
Procalcitonin (ng/mL)	0.14	2.56	-	3.19	-	8.31	-
SARS-CoV-2 nucleic acid	-	-	-	-	-	-	-
24-h urine volume (L)	0.86	0.15	0.12	0.05	0.10	0.15	0.13
Fever (°C)	38.3	38.5	39.0	38.7	38.9	38.8	38.9
Urine red blood cells (cells/HP)	++	++	-	+++	-	+++	-
Urine protein creatinine ratio (mg/g)	>300	>300	-	>300	-	>300	-
Abdominal pain	+	++	++	++	++	++	++

Subsequently, patient underwent emergency hemodialysis, renal needle biopsy, abdominal percutaneous puncture and drainage, and celiac lymph node biopsy on days 9, 10, 12, and 14.

**Figure 1.**
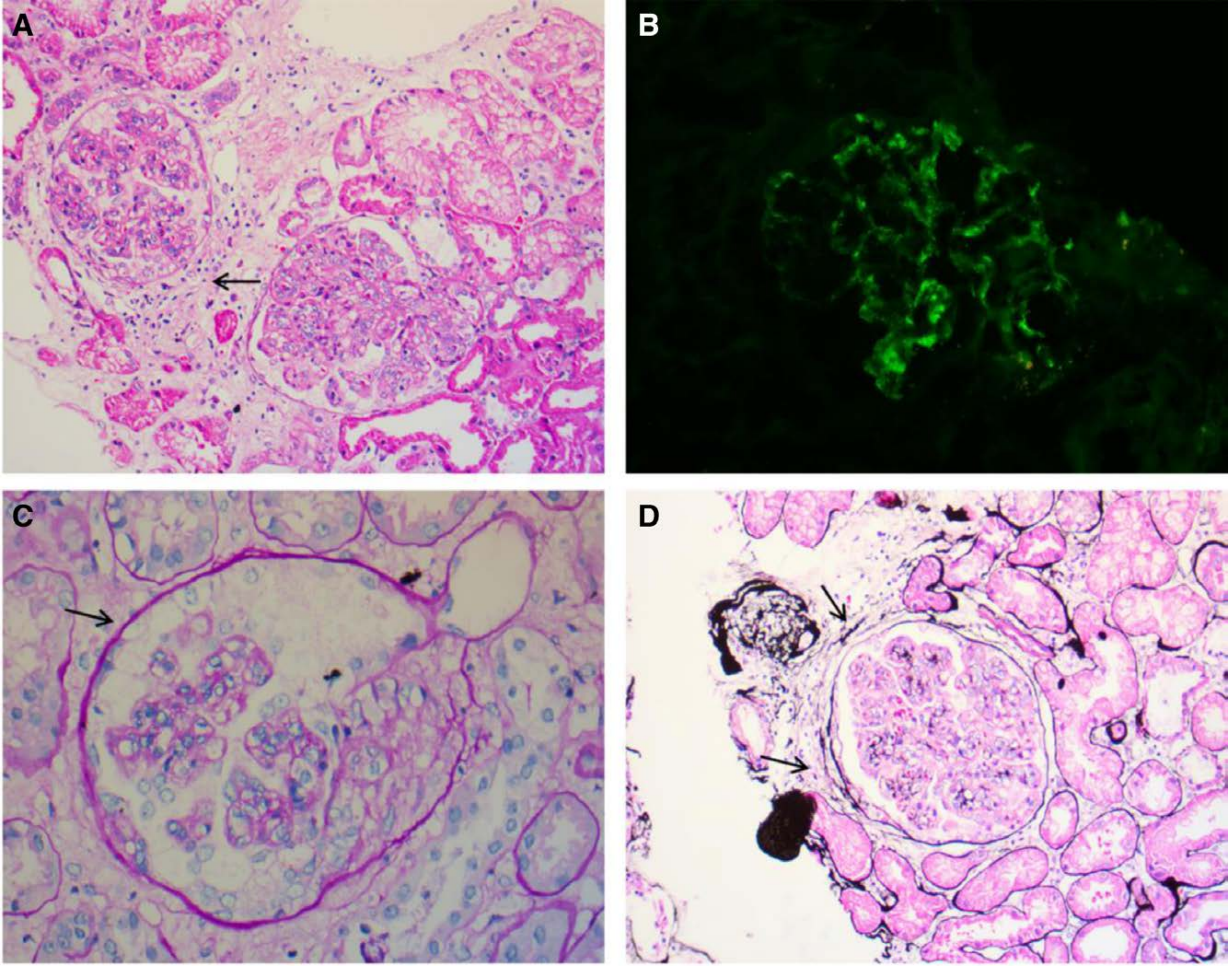
(A) (H and E, 200×) Llight microscopy showing diffuse endocapillary proliferative glomerulonephritis with cellular crescent formation (arrow). (B) (200×) Immunofluorescence showing bright mesangial and capillary loop staining for C3 (3+). (C) (PAS, 400×) endothelial swelling and neutrophil infiltration (arrow). (D) (PASM, 200×) fibrous crescents (arrows).

A renal biopsy was performed which revealed 26 glomeruli, 9 were cellular and fibrous crescents and 5 were globally sclerosed and the other had endocapillary hypercellularity. Immunofluorescence microscopy showed granular capillary loop staining for IgG (1+), IgG (3+), IgG (4+), IgM (1+), C3 (3+), C1q (1+), C4d (1+), and negative for IgA, PLA2R, and C4. Electron microscopy revealed glomerular endotheliosis with abundant infiltration cell inflammatory, and subendothelial electron-dense deposits (Fig. 1).

**Figure 2.**
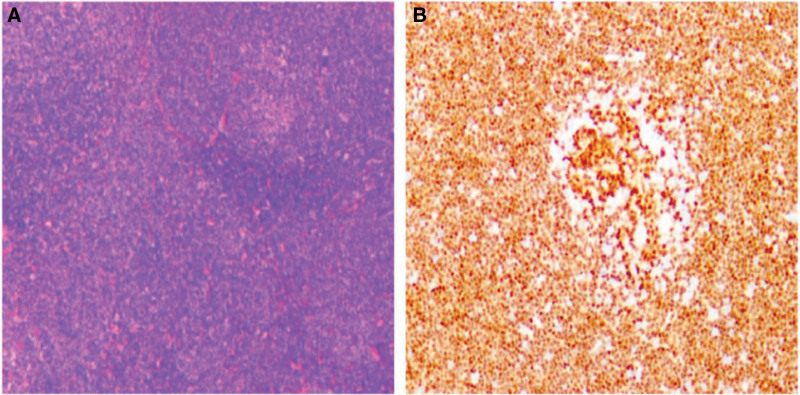
(A) (H and E, 200×) Diffuse infiltrate of monotonous small to medium-sized atypical lymphoid cells, and activated B cells in germinal centers. (B) (400×) The atypical lymphoid cells are CD20-positive.

Routine Intraperitoneal drainage fluid examination showed a large number of abnormal cells, which suspected lymphoma-like cells. Ultrasound-guided Celiac lymph node biopsy revealed the MCL with the positivity of CD5, CD20, CD23, cyclin D1, and Ki-67 of 25%, but negativity of CD3, CD15, CD30, and EBER (Fig. 2).

Furthermore, intraperitoneal drainage fluid was submitted to laboratory for bacterial, mycobacterial and fungal cultures and results were all negative. To detect which pathogen was involved, metagenomics next-generation sequencing (mNGS, using the Illumina NextSeq-500 Sequencing Platform, MATRIDX, Hangzhou, China) of abdominal drainage fluid was made. On day 17 of admission, the result showed that mycobacterium avium was positive with a higher relative abundance (57%) and the patient was diagnosed with PMAC.

**Figure 3.**
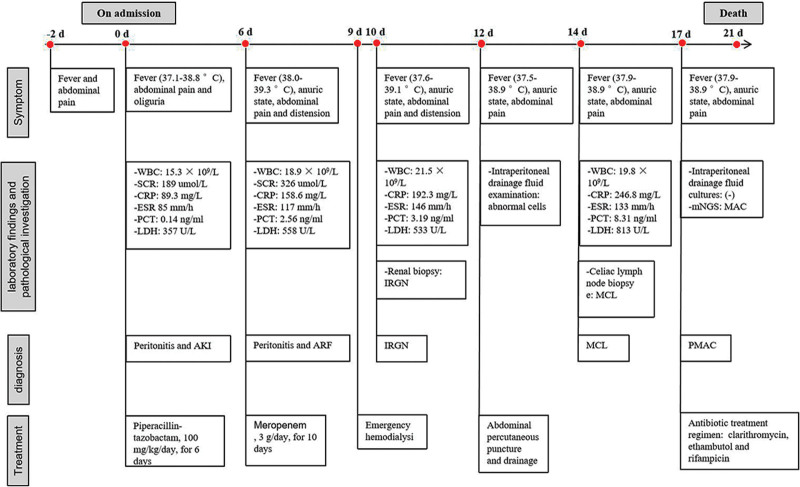
The entire process of diagnosis, treatment, and outcome.

Based on the results of the susceptibility tests provided by mNGS and considering the presence of PMAC in the patient, we adjusted new targeted antibiotic treatment regimen to clarithromycin, ethambutol and rifampicin. Unfortunately, the patient died of refractory septic shock after the 21^st^ day of admissiom. In addition, Figure 3 shown a timeline of thediagnosis and treatment.

## 3. Discussion and conclusion

Mycobacterium avium is a member of the NTM and a kind of opportunistic human pathogen, which is widespread in soil, water and other natural environments, and mainly Invading the host body through the respiratory tract, the gastrointestinal tract or the skin.^[10]^ MAC usually infects patients with immunocompromised state including HIV, peritoneal dialysis, organ transplantation, and tumor. When the immune system was damaged, patients with non-HIV or without underlying disease had a clearly increased risk of MAC infection. In addition to pneumonia, MAC often causes infections of extrapulmonary sites, such as bone, liver, spinal cord, intestinal tract, skin, and pericardium, Whereas PMAC was rarely mentioned. To date, 51 cases of PMAC have been reported, including only 1 PMAC case with tuberculosis infection before developed IRGN and 2 peritoneal dialysis cases developed PMAC. To the best of our knowledge, MCL and IRGN in the same patient caused by PMAC have not been reported. Here, we reported the first case of MCL and IRGN caused by PMAC.

The diagnosis of MAC infection mainly relies on clinical, microbiological, and imaging criteria. Since the symptoms and imaging manifestations of mac are similar to Mycobacterium tuberculosis, the diagnosis of mac mainly depends on bacterial culture. However, the positive rate of bacterial culture for mac is low, and it usually takes up to 6 weeks of culture to show signs of growth, so early diagnosis of MAC infection is a difficult challenge. In our case, multiple ascites cultures of the ascites were negative and finally relied on mNGS to diagnose MAC. Notably, mNGS can simultaneously detect various types of potential pathogens with rapid speed, high sensitivity and high specificity. In our case, MAC was not cultured in intraperitoneal drainage fluid, but detected by mNGS within only 2 days. This indicates that mNGS is more favorable to identify some rare or unknown pathogens than traditional microbiological detection methods.

An optimal antimicrobial treatment regimen for PMAC have not been firmly established. According to the recommendations of international clinical guidelines and consensus on NTM pneumonia,^[11–13]^ the standard treatment of PMAC includes macrolides, rifamycin, and ethambutol. Some clinical studies have shown that a 2-drug regimen containing clarithromycin and ethambutol can achieve the same effect as a 3-drug combination therapy for MAC.^[6]^

However, this is not recommended by guidelines, possibly due to the fact in the studies mentioned above that the patient had mild disease and a low rate of deterioration. Duration of PMAC treatment is also not clearly defined or consistent but is recommended for at least 12 months, depending on the patient’s tolerance, underlying diseases and immune function.^[14]^ Shunsuke et al^[15]^ reported that a 4-year-old peritoneal dialysis (PD) boy with PMAC was promptly removed from the PD catheter and received hemodialysis with a cuff catheter inserted through the internal jugular vein while being treated with clarithromycin, ethambuterol, and rifampicin, and successfully underwent a kidney transplant at 20 months. The above cases emphasizes that dialysis catheter removal is key to cure MAC infection in PD patients. After the diagnosis of PMAC, the patient unfortunately died within days of the classic 3-drug combination regimen, and the direct cause of death was attributed to septic shock.

Furthermore, IRGN and MCL ccould confirm the diagnosis. In our case, a debate may arise about any causal relationship between IRGN, MCL, and PMCA. Mary Rithu Varkey et all.^[16]^ reported a case of a immunocompetent patients with disseminated MAC pneumonia, which failed to respond to 1 year of guideline-based antibiotic therapy and was diagnosed as Hodgkin lymphoma by lymph node biopsy. It is unknown whether disseminated MAC preceded lymphoma or early undiagnosed lymphoma caused MAC pneumonia. Chen Shujuan et al^[17]^ showed a case of RPGN caused by MAC pulmonary, and renal function recovered after only 12 months of anti-NTM therapy. We strongly believe that MAC infection is the basic reason for the occurrence of IRGN and MCL. Firstly, the time to onset was significantly earlier in PMAC than in IRGN and MCL; Second, the renal biopsy showed diffuse endothelial cell proliferation with neutrophil exudation as a typical characteristic feature of IRGN^[18]^; Third, MCL is more common in elderly patients, and its onset is mostly related to infection and immune dysfunction.^[19]^ However, it is not clear whether there is an association between IRGN and MCL in the present case. With regard to the treatment of IRGN and MCL, we expected that Anti-mac treatment regimen may be effective to manage these complications. After the diagnosis of PMAC, we chose the classic 3-drug combination regimen, but the patient eventually died due to the aggravation of infection. We speculate that it may be MCL as well as associated with drug side effects that increase the difficulty of MAC treatment.

In the current case, this old patient underwent a protracted and intricate process of diagnosis spanning over 17 days. The Final diagnosis of PMAC, MCL, and IRGN were established based on the clinical manifestations, the results of laboratory, mNGS, imaging examinations and biopsy. In respect of therapeutic options, we first choose empiric antimicrobial and then changed to diagnostic anti-MAC treatment while ignoring the treatment of MCL. This case illustrates the difficulties in diagnosing and treating an immunocompetent patient with MCL and IRGN caused by PMAC.

We report a rare and intricate case of MCL and IRGN caused by PMAC. Our report adds to the literature on complications of MAC, which rarely occur in immunocompetent people. Early diagnosis and personalized treatment of PMAC and its complications are challenging, and more researches are needed to determine the best diagnosis and treatment regimen for patients with PMCA in future. In addition, mNGS should be considered in patients with unexplained infections and culture-negative infection to ensure timely and prompt diagnosis and treatment.

## Author contributions

**Data curation:** Yiqi Huang, Weigang Shen.

**Formal analysis:** Li Xia.

**Supervision:** Tianxiao Fu.
